# Optimal Design of High-Critical-Current SMES Magnets: From Single to Multi-Solenoid Configurations

**DOI:** 10.3390/ma18194567

**Published:** 2025-10-01

**Authors:** Haojie You, Houkuan Li, Lin Fu, Boyang Shen, Miangang Tang, Xiaoyuan Chen

**Affiliations:** 1 School of Engineering, Sichuan Normal University, Chengdu 610101, China; 2022180148@stu.sicnu.edu.cn (H.Y.); chenxy@sicnu.edu.cn (X.C.); 2 School of Economics and Management, Sichuan Normal University, Chengdu 610101, China; litchki@stu.sicnu.edu.cn; 3 College of Transportation, Tongji University, Shanghai 201804, China; shenboyang@tongji.edu.cn; 4 Sichuan Energy Internet Research Institute, Tsinghua University, Chengdu 610101, China

**Keywords:** superconducting magnetic energy storage (SMES), superconducting magnet design, adaptive genetic algorithm, critical current anisotropy, air gap optimization

## Abstract

Advanced energy storage solutions are required to mitigate grid destabilization caused by high-penetration renewable energy integration. Superconducting Magnetic Energy Storage (SMES) offers ultrafast response (<1 ms), high efficiency (>95%), and almost unlimited cycling life. However, its commercialization is hindered by the complex modeling of critical current with anisotropic behaviors and the computational inefficiency of high-dimensional optimization for megajoule (MJ)-class magnets. This paper proposes an integrated design framework synergizing a two-dimensional axisymmetric magnetic field model based on Conway’s current-sheet theory, a critical current anisotropy characterization model, and an adaptive genetic algorithm (AGA). A superconducting magnet optimization model incorporating co-calculation of electromagnetic parameters is established. A dual-module chromosome encoding strategy (discrete gap index + nonlinear increment) and parallel acceleration techniques were developed. This approach achieved efficient optimization of MJ-class magnets. For a single solenoid, the critical current increased by 22.6% (915 A) and energy storage capacity grew by 41.8% (1.12 MJ). A 20-unit array optimized by coordinated gap adjustment achieved a matched inductance/current of 0.15 H/827 A (20 MJ), which can enhance transient stability control capability in smart grids. The proposed method provides a computationally efficient design paradigm and user-friendly teaching software tool for high-current SMES magnets, supporting the development of large-scale High-Temperature Superconducting (HTS) magnets, promoting the deployment of large-scale HTS magnets in smart grids and high-field applications.

## 1. Introduction

The high-penetration integration of renewable energy sources exacerbates dynamic instability in modern power systems. The large-scale integration of intermittent energy sources like wind and photovoltaics intensifies grid fluctuations [1]. Conventional energy storage technologies are constrained by response speed (e.g., battery response time ~100 ms) and cycle life (typically below 10,000 cycles) [2], making them inadequate for millisecond-level power quality management requirements. Superconducting Magnetic Energy Storage (SMES), with its ultrafast response (<1 ms), ultra-high efficiency (>95%) [3], and almost infinite charge/discharge cycle life, emerges as an ideal solution for suppressing grid transient disturbances [4].

However, the capital cost of superconducting energy storage magnets is relatively high. It is necessary to optimize the design of their internal magnet structure to obtain the targeted energy storage capacity and current rating required by the power grid at the lowest economic cost. The energy storage capacity of a magnet is directly proportional to its inductance and the square of the critical current.

Due to the anisotropy of superconductors, the critical current degradation rate of REBCO tapes under perpendicular magnetic fields can be several times higher than under parallel fields [5,6]. Traditional design methods attempt to accurately quantify the coupled influence of the magnetic field vector on critical current [7]. Multi-parameter co-optimization of MJ-class magnets constitutes a high-dimensional nonlinear constrained problem [8]. While the Finite Element Method (FEM) offers high accuracy, a single full-parameter simulation can take several hours, rendering it impractical for iterative optimization. Although Simulated Annealing (SA) has been applied to magnet structural optimization, its convergence efficiency is low, and engineering adaptability is weak [9].

For the analytical optimization solutions, Conway’s analytical method for rectangular cross-section coil fields laid the foundation for efficient field computation, but this model did not incorporate the anisotropic properties of superconducting materials [10]; Hajiri et al. introduced an electromagnetic-thermal finite element model coupled to a reduced electrical circuit to simulate quench propagation in inductive HTS coils under overcurrent regimes. By incorporating flux-flow resistance and contact resistance effects, the model enhances quench prediction accuracy; however, it is limited to single-coil analysis and ignores material anisotropy in current distribution [11]. Li et al. focused on AC loss optimization in HTS SMES integrated with hydrogen-battery hybrid systems. Their work synergizes energy management strategies with magnet AC loss reduction, improving system-level efficiency, but relies on simplified axisymmetric models that cannot accurately capture 3D fringe fields and omits mechanical stress effects on critical current degradation [12]. Notably, Xue et al. pioneered a large-scale GPU-optimized algorithm to resolve coupled electromagnetic-thermal-strain dynamics during quenching in multifilamentary Nb_3_Sn coils. Their model uncovered the correlation between instability propagation velocity and cumulative Joule heating (rather than instantaneous power), offering key insights for magnet design. However, this framework remains restricted to Nb_3_Sn materials and does not address the critical current anisotropy inherent in REBCO tapes [13]. These limitations result in existing magnets having low energy density and insufficient tape utilization, severely constraining the economic viability of SMES magnets.

Large-scale superconducting magnet systems (such as the ITER Tokamak [14], CERN’s Large Hadron Collider (LHC) [15], and China Southern Power Grid’s 10 MJ SMES demonstration project [16]) have comprehensively addressed engineering feasibility issues in their designs. These projects have employed advanced technologies such as forced-flow helium cooling, improved mechanical support, and active quench protection system combining energy extraction and segmentation. For instance, the ITER project utilizes large toroidal magnet systems with carefully designed mechanical support structures to cope with enormous Lorentz forces and employs multi-level protection systems to ensure safe operation. Similarly, LHC’s superconducting rings employ distributed cooling systems and advanced quench detection technologies to manage high energy storage capacity (approximately 700 MJ).

However, these pioneering systems were primarily optimized for their specific scientific or demonstration objectives, often prioritizing extreme performance metrics over magnet design economy and scalability. This has left a gap in systematic methodologies that simultaneously address electromagnetic performance, geometric constraints, and practical manufacturability for commercial energy storage applications.

To overcome these challenges, this article proposed a SMES magnet design methodology synergizing analytical model and intelligent algorithms. First, a two-dimensional axisymmetric magnetic field model for pancake coils was established based on Conway’s current-sheet theory, incorporating the critical current anisotropy to quantify the electro-magnetic coupling behavior. Second, targeting two Non-deterministic Polynomial-time hard problems (NP-hard problems [17]) of magnet dimensional optimization and inter-pancake gap distribution, an adaptive genetic algorithm was developed to feature dual-module chromosome encoding (discrete gap indexing + stochastic increment seeding), elite preservation strategy, adaptive mutation, and accelerated computation via vectorization and parallel processing. Furthermore, the optimized single-solenoid unit was extended to a parallel magnet array comprising multiple solenoid units, where coordinated optimization of the inter-unit gaps was implemented to achieve precise inductance matching and minimize electromagnetic coupling interference.

## 2. Methodology

### 2.1. Magnetic Field Calculation

**Figure 1:**
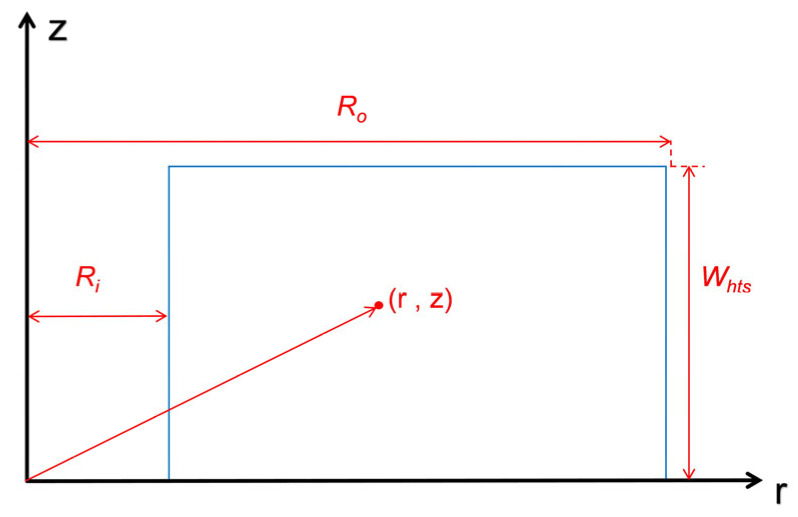
Schematic diagram of a single pancake coil.

Based on Conway’s current-sheet theory [10], a 2D analytical solution for the magnetic field generated by pancake coils is established. For a single pancake coil with inner radius *R_i_*, outer radius *R_o_*, and height *W_hts_*, as shown in Figure 1, the magnetic field at a point (*r,z*) in cylindrical coordinates can be decomposed into:

Axial component (parallel to the tape surface):

(1) $$B_{∥}\left(r,z\right)=\mu_{0}J\left(R_{o}-r\right)-\psi_{1}\left(R_{i},R_{o},r,\left|z\right|\right)-\psi_{1}\left(R_{i},R_{o},r,\left|W_{hts}-z\right|\right)$$

Radial component (perpendicular to the tape surface):

(2) $$B_{⊥}\left(r,z\right)=\psi_{2}\left(R_{i},R_{o},r,\left|W_{hts}-z\right|\right)-\psi_{2}\left(R_{i},R_{o},r,\left|z\right|\right)$$

The simplified forms of the core functions *ψ*_1_ and *ψ*_2_ are given in Equations (3) and (4). The complete mathematical descriptions, encompassing the full set of integral terms *L*_1_ and *L*_2_, are provided in Appendix A (Equations (A1) and (A2)).

(3) $$\psi_{1}\left(R_{i},R_{o},r,a\right)=\frac{\mu_{0}J}{2}\left(R_{o}-R_{i}\right)-\frac{\mu_{0}Ja}{2}ln\left(\frac{R_{o}+r+\sqrt{{\left(R_{o}+r\right)}^{2}}+a^{2}}{R_{i}+r+\sqrt{{\left(R_{i}+r\right)}^{2}\;}+a^{2}}\right)+L_{1}({R_{i},R}_{o},r,a)$$

(4) $$\psi_{2}\left(R_{i},R_{o},r,a\right)=\frac{\mu_{0}Jr}{4}ln\left(\frac{R_{o}+r+\sqrt{{\left(R_{o}+r\right)}^{2}+a^{2}}}{R_{i}+r+\sqrt{{\left(R_{i}+r\right)}^{2}+a^{2}}}\right)+L_{2}({R_{i},R}_{o},r,a)$$

where the geometric factor $X\left(R,r,a,\theta \right)$ is

(5) $$X\left(R,r,a,\theta \right)=\sqrt{R^{2}+r^{2}+a^{2}-2Rrcos\theta },$$

*J* is the current density per unit volume, *µ*_0_ is the vacuum permeability (4π × 10^−7^ H m^−1^), and *a* represents |*z*| or |*W_hts_* − *z*|.

### 2.2. Critical Current Anisotropy Model

**Figure 2:**
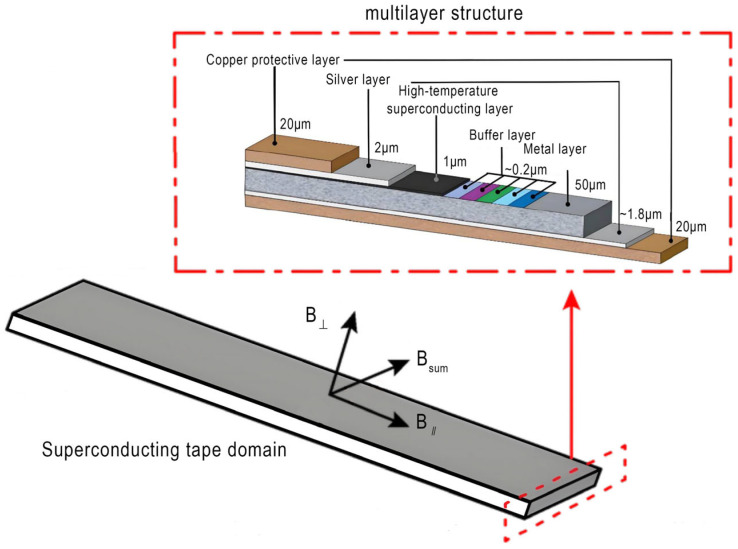
Schematic diagram of REBCO superconducting tape.

REBCO superconducting tapes exhibit field angle dependence in their critical current *I_c_* [18,19]. They achieve high-current transmission in strong fields at liquid-nitrogen temperatures through heteroepitaxial integration on flexible metal substrates, nano-buffer layers [20,21], REBCO superconducting films, and Ag/Cu composite stabilizing layers [20]. The structure of the REBCO superconducting tape is shown in Figure 2. The critical current is calculated as follows:

(6) $$I_{c}\left(B_{∥},B_{⊥}\right)=I_{c0}\times {\left(1+\frac{∥B{∥}_{eff}}{B_{1}}\right)}^{-\alpha }$$

where the effective magnetic field $∥B{∥}_{eff}$ is:

(7) $$∥B{∥}_{eff}=\sqrt{{\left(\gamma^{-1}B_{∥}\right)}^{2}+B_{⊥}^{2}}$$

**Figure 3:**
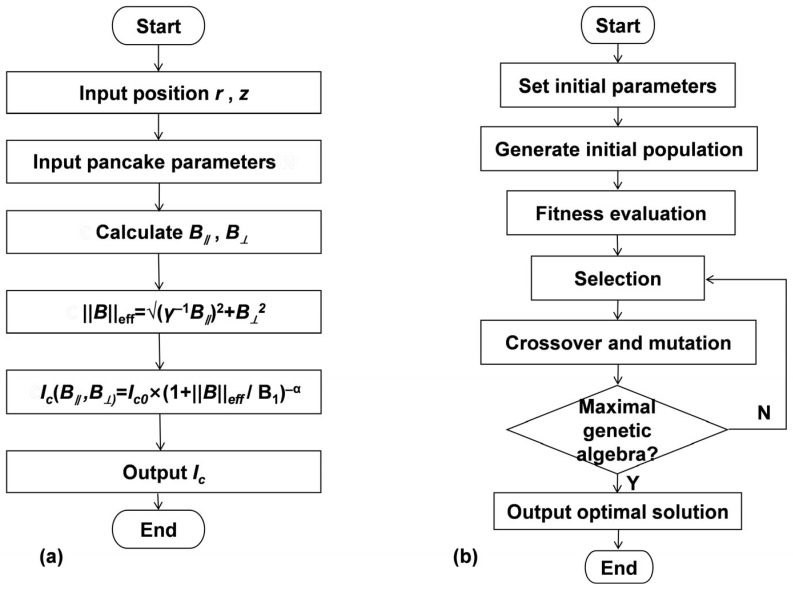
Model calculation and optimization framework. (**a**): Critical current calculation flowchart; (**b**): Adaptive genetic algorithm structure.

Experimentally fitted parameters (20 K operation): *I_c_*_0_ = 1736 A (zero-field critical current) [9,21,22,23], *B*_1_ = 1.59 T (characteristic magnetic field), γ = 3.38 (anisotropy ratio), *α* = 0.88 (decay exponent) [24,25,26]. The parameters *I*_c0_, *B*_1_, *γ*, and *α* were adopted from experimental characterizations of commercial REBCO tapes [9,18,26,27], ensuring the model’s relevance to practical HTS magnet design. The critical current calculation flowchart is shown in Figure 3a.

### 2.3. Inductance Calculation Model

Utilizing self-inductance and mutual inductance theory [28], the self-inductance of a single pancake is calculated as follows:

(8) $$L_{self}=\frac{\pi \mu_{0}R_{i}N^{2}}{16\varphi }\cdot \frac{{\left(\tau \;+\;1\right)}^{2}}{1\;+\;0.9\frac{\tau \;+\;1}{4\varphi }\;+\;0.64\frac{\tau \;-\;1}{\tau \;+\;1}\;+\;0.84\frac{\tau \;-\;1}{2\varphi }}$$

where τ = *R_o_*/*R_i_* (radius ratio), φ=W*_hts_*/2*R_i_* (form factor)

Pancake-to-pancake mutual inductance is calculated as follows [29,30]:

(9) $$M_{ij}=\frac{1}{2}\left(L_{i+j}+L_{gap}-L_{i}-L_{j}\right)$$

where *L_i+j_* is the equivalent self-inductance when pancake coils *i* and *j* are merged, *L_gap_* denotes the inductance component contributed by the air-gap region between the two coils, *L_i_* and *L_j_* are the self-inductances of the individual pancake coils *i* and *j*, respectively.

The total inductance of a multi-pancake system is:

(10) $$L_{total}=\sum_{k=1}^{Q}L_{k}+2\sum_{i=1}^{Q-1}\sum_{j=i+1}^{Q}M_{ij}\cdot C_{ij}$$

where the coupling coefficient $C_{ij}=e^{-\kappa \left|z_{i}\;-{\;z}_{j}\right|},\;\kappa$ is an empirical decay factor.

### 2.4. Genetic Algorithm Optimization Design

For the single solenoid size optimization and single solenoid gap optimization problems, the constraints are defined as follows:

(11) $$E_{max}=\frac{1}{2}L_{sum}I_{c}^{2}\;s.t.\;\left\{\begin{array}{ll} \pi \left(W_{turn}N+2R_{i}\right)NQ\leq S_{sum} & \\ R_{o}=R_{i}+W_{turn}N\leq q & \\ I_{c}\geq I_{op} & \end{array}\right.$$

where *N* is the number of turns per pancake, *W_turn_* is the equivalent single-turn width (including insulation), *L_sum_* is the total inductance (including self and mutual inductance), *E_max_* is the maximum energy storage capacity, *q* is the maximum outer radius per pancake, *I_c_* is calculated by the anisotropic critical current model using Equation (6), and *I_op_* is the minimum operating current requirement of the magnet.

(12) $$\left\{\begin{array}{ll} g\in \left[g_{1},g_{2}\right] & \\ m\leq n & \\ S_{sum}\leq p & \\ \left|I_{c,calc}-I_{c,actual}\right|/I_{c,actual}\leq 0.01 & \\ \forall \;positon\;k:\;left\;spacing\;g_{k}=right\;spacing\;g_{N-k+1} & \end{array}\right.$$

where *g* is the starting gap spacing, *g*_1_ is its lower limit, *g*_2_ is its upper limit, *m* is the number of movable pancakes on one side, *n* is the total number of pancakes on one side, *S_sum_* is the total tape length, *p* is the maximum allowable total tape length, and *I_c,calc_* is the calculated critical current value.

Targeting the two NP-hard problems defined by Equation (11) (size) and Equation (12) (gaps), and considering the unknown structure of their solution space, an improved GA framework is proposed in Figure 3b and described as follows

(1) Dual-Module Chromosome Encoding

Module 1 uses a discretized gap value index (10-bit gene, mapping to 1024 candidate points). Module 2 uses a random seed to control the mirror increment Δ*x*, enabling a nonlinear distribution of gap spacings for pancakes moved on one side:

(13) gap=g+Δx

where g ∈ $\left[g_{1},g_{2}\right]$ randomly selected per calculation.

(14) Δx=β×g

where β ∈ [0, 1],Δx ∈ $\left[0,g_{2}\right]$

(2) Adaptive Mechanism

Mutation probability is dynamically adjusted based on individual fitness [31,32]: decreased for high-fitness individuals and increased for low-fitness individuals. An elitist preservation strategy maintains population diversity.

(3) Acceleration Strategies

Vectorized analytical magnetic field computation, memory pre-allocation to reduce dynamic overhead, and parallel evaluation of population fitness are implemented. This approach significantly enhances the solving efficiency and accuracy of high-dimensional constrained problems by decoupling the variable space and employing intelligent encoding strategies [33].

## 3. Optimization Strategy and Framework

This study employed a phased optimization strategy to design a high-energy-density SMES solenoid magnet:

(1) Single Solenoid Size Optimization

**Figure 4:**
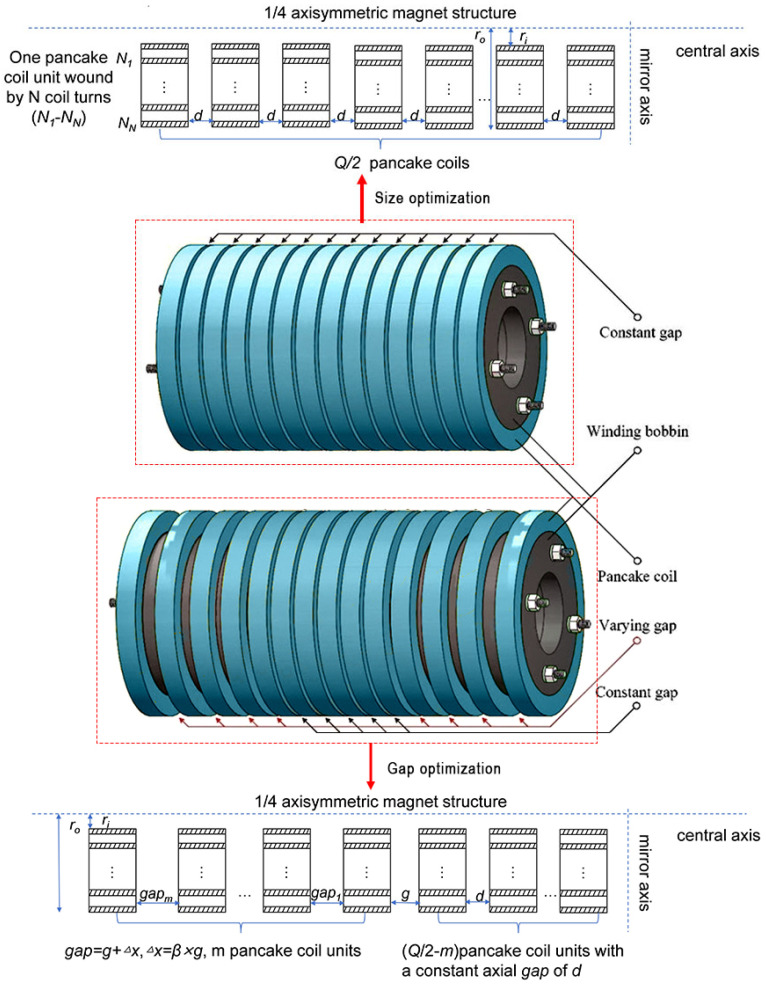
Schematic diagram of single solenoid size optimization and single solenoid gap distribution optimization.

Under the constraints of Equation (11) (including maximum tape length *S_sum_*, maximum pancake outer radius *q*, and minimum operating current *I_op_*), with the goal of maximum energy storage capacity *E_max_*, the key decision variables were the inner radius *R_i_* and the number of pancakes *Q*. As shown in Figure 4, this step determined the optimal *R_i_*, *R_o_*, total height *H*, total pancakes *Q*, turns per pancake *N*, and the corresponding inductance *L_sum_* and critical current *I_c_*.

(2) Single Solenoid Gap Distribution Optimization

Based on the optimized configuration from Step 1, under the constraints of Equation (12) (including number of movable pancakes per side *m*, gap spacing *g*), gap distribution optimization is carried out. As shown in Figure 4, the two variables were *m* and *g*. A specific encoding strategy searched for the optimal gap to further enhance *E_max_*. The output results include the optimal *m*, gap sequence, inductance *L*, critical current *I_c_,* and tape usage *S*.

(3) Multi-Solenoid Parallel Array

**Figure 5:**
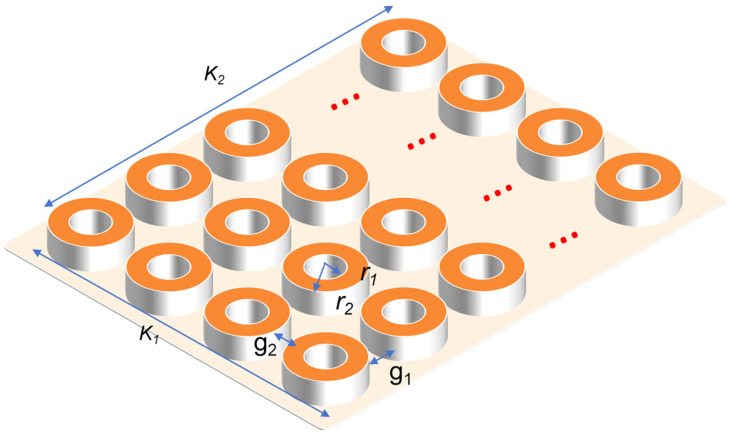
Schematic diagram of multi-solenoid parallel array.

Based on the optimized single-solenoid unit derived from the above two steps, a parallel array comprising multiple units is constructed to achieve the targeted energy storage capacity. This step involves the coordinated optimization of horizontal and vertical inter-unit gaps. This step involves the coordinated optimization of the inter-unit gaps in the x-direction (*g*_1_) and y-direction (*g*_2_). The primary objective is to maximize the system’s energy storage capacity while rigorously ensuring the convergence of the total inductance to a specified value, thereby establishing a comprehensive design framework for large-scale SMES magnets characterized by low coupling interference. Figure 5 is a schematic diagram of multi-solenoid parallel array.

## 4. Optimization of the Single-Solenoid

### 4.1. Single Solenoid Size Optimization

Aiming to maximize energy storage capacity under a fixed tape length constraint (*S_sum_* = 5 km), key geometric parameters were optimized simultaneously based on the adaptive genetic algorithm. The inner radius *R_i_* range was set to 300–500 mm, and the number of pancakes *Q* was set between 20–100. Other parameters are listed in Table 1. The constraints for the fitness evaluation are given by Equation (11).

**Figure 6:**
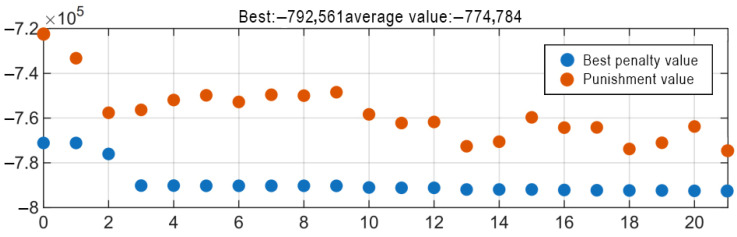
Visualization analysis of GA iteration process and variable optimization results.

The genetic algorithm optimization are shown in Figure 6. The maximum energy storage capacity of 0.79 MJ (corresponding to the minimum objective function value of −0.79 × 10^6^ J) was achieved with 87 pancakes, an inner radius of 0.48 m, an outer radius of 0.49 m, 19 turns per pancake, and a total height of 0.43 m. The corresponding critical current was 747 A, and the inductance was 2.84 H.

### 4.2. Single Solenoid Gap Optimization

In the second step, gap optimization of the single solenoid was performed. Based on the optimal result from Step 1 (87 pancakes, 19 turns per pancake, inner radius 0.48 m, outer radius 0.49 m, total height 0.43 m), a dual-module optimization strategy was employed: Module 1 established a discretized search space for gap spacing. The feasible domain for the starting gap spacing *g* was set to [0, 2 mm], uniformly divided into 1024 candidate points (controlled by a 10-bit chromosome length), forming a high-precision discrete solution space. Module 2 generated random seed values, which are used to produce axial position adjustment increments, causing adjacent gap increments to increase nonlinearly and randomly. According to Equations (13) and (14), with *g* ∈ [0, 2 mm] and Δx ∈ [0, 2 mm], a nonlinear distribution of gap spacings among pancakes can be realized.

The constraints for the fitness evaluation in the calculation process are given by Equation (12). During fitness evaluation, the algorithm reconstructed the axial distribution of the coil pancakes based on the decoded gap parameters and position increments. The 2D magnetic field distribution was calculated using the analytical model, followed by the critical current *I_c_* and inductance *L*, with energy storage capacity serving as the optimization objective function. An elitist preservation strategy maintained population diversity during evolution, driven by tournament selection, crossover, and mutation operations.

The optimization framework adopted a two-layer iterative structure: the outer loop traversed candidate values for the number of movable pancakes per side *m*, while the inner loop jointly optimized the gap spacing *gap* and position adjustment increment using the genetic algorithm. This decoupled optimization strategy ensured variable synergy while significantly reducing computational complexity.

**Figure 7:**
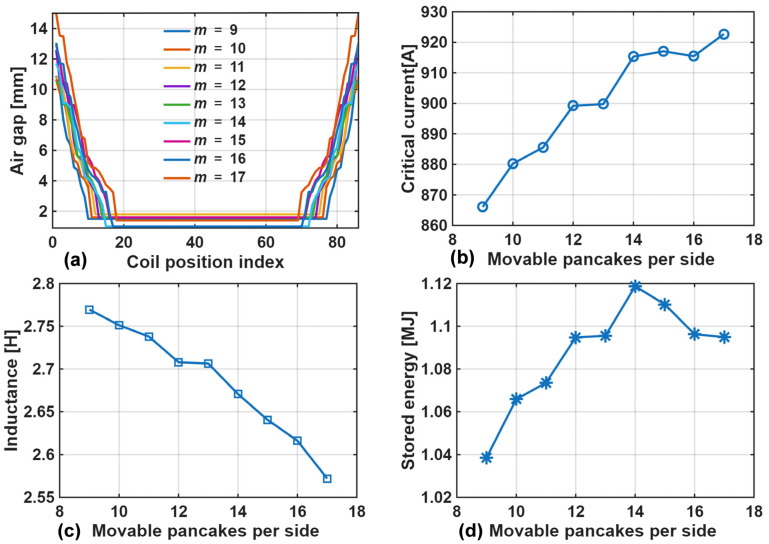
Performance enhancement of single solenoid via gap op: (**a**) Air-gap distribution; (**b**): Critical current versus number of movable pancakes per side; (**c**): Inductance versus number of movable pancakes per side; (**d**): Stored energy versus number of movable pancakes per side.

The results for critical current, inductance, stored energy, and gap spacing are shown in Figure 7 and Table 2. The results indicate that the maximum energy storage capacity of 1.12 MJ was achieved when the number of movable pancakes per side was 14. The corresponding inductance was 2.67 H, critical current was 915 A, and tape usage was 4810 m.

Compared to typical superconducting modeling methods [34,35,36], the proposed intelligent optimization scheme substantially reduces computational time while preserving physical model accuracy. It achieves concurrent enhancements of 41.8% in energy storage capacity and 22.6% in critical current, delivering an efficient paradigm for large-scale HTS magnet design.

## 5. Optimization of the Multi-Solenoid Parallel Configuration

### 5.1. Multi-Solenoid Modeling

Based on the optimization results from the previous two steps, the design was extended to the optimization of a multi-solenoid parallel configuration. To meet the high-power compensation requirements in large-capacity microgrid systems, the superconducting magnet requires a very high critical current [37,38]. This paper employs solenoid magnets to design a 20 MJ-class SMES magnet. Since a single solenoid magnet cannot meet the 10 kA-class operating current requirement, a parallel configuration was introduced, consisting of 20 parallel-connected MJ-class superconducting solenoid coil units.

Figure 5 shows the parallel matrix diagram of multiple solenoids. The overall structure consists of a 4 × 5 solenoid matrix array. The inner and outer radii of each solenoid unit are *r*_1_ and *r*_2_, respectively. The gap between adjacent solenoid units in the horizontal and vertical directions are *g*_1_ and *g*_2_, respectively. The gap between adjacent solenoid units in the x-direction and y-direction are set as *g*_1_ and *g*_2_, respectively. The overall width *K*_1_ of the magnet is 8*r*_2_ + 3*g*_2_, and the length *K*_2_ is 10*r*_2_ + 4*g*_1_. For the 2.67 H/915 A/1.12 MJ superconducting solenoid coil, *r*_1_ and *r*_2_ are 0.48 m and 0.49 m, respectively.

For high-power compensation applications in microgrids, the 4 × 5 solenoid matrix array (20 units) needs to maintain an ultra-high critical current of 18 kA. However, the inherent electromagnetic anisotropy couples with the mutual inductance effects between adjacent coil units [39,40]. Insufficient coil spacing leads to superimposed mutual magnetic fields significantly enhancing the local field, resulting in degradation of the critical current [41,42]. To meet the stringent targets of 20 MJ stored energy and 0.13 H total inductance, coordinated optimization of the gap parameters is required to achieve the dual objectives of inductance and critical current. Given that meter-scale inter-unit gaps dominate the solenoid matrix array’s geometry, electromagnetic coil interaction forces are negligible within the electromagnetic optimization framework of this study.

All 20 coil units are solenoids with parallel central axes. The mutual inductance among any two solenoids is calculated as follows [43]:

(15) $$M=\frac{\pi \mu_{0}D^{3}\Omega^{2}\xi }{{16h}^{2}}\times \left[\begin{array}{l} \frac{c}{z}-1+\frac{1}{2}\xi^{2}(1-\frac{3}{2}\eta^{2}+\frac{1}{2\eta^{3}})-\frac{5}{8}\xi^{4}(1-5\eta^{2}+\frac{35}{8}\eta^{4}-\frac{3}{8\eta^{5}})\\ +\frac{35}{32}\xi^{6}(1-\frac{21}{2}\eta^{2}+\frac{189}{8}\eta^{4}-\frac{231}{16}\eta^{6}+\frac{5}{16}\eta^{7})+\ldots \end{array}\right]$$

Ω is the number of turns per solenoid; *D* is the average diameter of the solenoid; *h* is the height of the solenoid; *z* is the distance between the axes of the two solenoids; the structural parameters *c* = (*z*^2^ + *h*^2^)^1/2^, *ξ* = *D*/(2*h*), *η* = *z*/*c*.

Starting from the first-row solenoid, the units are sequentially defined from left to right as coil unit 1, 2, …, 20. The total inductance *L_i_* of the *i*th coil unit is calculated as:

(16) $$L_{i}=L_{0}+\sum_{j=1}^{j=19}M_{ij}$$

*L*_0_ is the self-inductance of the *i*th coil unit; *M_ij_* is the mutual inductance among the *i*th coil unit and the remaining 19 coil units. It should be noted that since the structural parameters of all the 20 coil units are identical, the self-inductance of each unit is *L*_0_.

The total inductance *L*_total_ of the entire magnet is then calculated as:

(17) $$L_{total}=\sum_{k=1}^{20}L_{0}+2\sum_{i=1}^{19}\sum_{j=i+1}^{20}M_{ij}$$

### 5.2. Optimized Results

**Figure 8:**
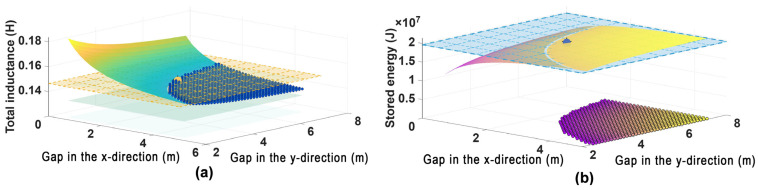
Dependence of the multi-solenoid array performance on the inter-unit gaps: (**a**) total inductance; (**b**) stored energy.

The co-optimization results of the multi-solenoid array are presented in Figure 8. Figure 8a quantifies the nonlinear dependence of the total inductance, *L*_total_, on the inter-unit gap in the x-direction, *g*_1_, and the inter-unit gap in the y-direction, *g*_2_. The inductance exceeds the target value of 0.13 H for *g*_1_ < 2.5 and *g*_2_ < 4.5 m due to the strong magnetic coupling. Conversely, it decays below the target value when *g*_1_ > 3.0 m or *g*_2_ > 5.0 m, indicating very weak mutual inductance. The yellow grid surface in Figure 8a corresponds to the actual inductance value of 0.15 H at the global optimum. The overall dimensions of the optimized 20-unit array are approximately *K*_1_ = 18.2 m in width, and *K*_2_ = 24.0 m in length, with a height of 0.74 m per solenoid.

Figure 8b presents the relationships among the energy storage capacity and the two gaps. The peak energy storage of 20 MJ is achieved at the optimal gap configuration of (*g*_1_, *g*_2_) = (2.44, 4.77)  m. At this point, the total inductance is 0.15 H, and the critical current per unit is 827 A, representing a slight degradation of approximately 9.7% from the initial single-unit value. The blue grid surface marks ±10% tolerance range around the target inductance (0.13 H), i.e., 0.12 H ≤ *L*_total_ ≤ 0.14 H. Analysis of the data points within this range (scatter region) confirms an approximately linear relationship between the inductance deviation (Δ*L*) and the critical current degradation ratio (Δ*I*_c_/*I*_c0_).

## 6. Conclusions

This study proposed a systematical methodology to overcome challenges in the design of large-scale SMES magnets. An analytical magnetic field computation framework integrating REBCO’s critical current anisotropy was established, addressing the limitation of conventional methods which neglect magnetic field vector coupling effects. An inductance calculation scheme incorporating exponentially decaying coupling coefficients was developed, substantially enhancing computational efficiency in multi-pancake systems.

To address high-dimensional nonlinear constrained problems, a dual-module genetic algorithm (discrete gap index + random increment encoding) was designed. Combined with an adaptive mutation strategy and parallel computation, it enhanced the optimization efficiency for MJ-class magnets by two orders of magnitude. In gap distribution optimization, by decoupling the variable space and employing a hierarchical iteration strategy, an increase of 22.6% in critical current and 41.8% in energy storage capacity was achieved.

The optimized single solenoid achieved an energy storage density of 1.12 MJ. The innovative coordinated gap design for the 20-unit (4 × 5) parallel array maintained the unit current at 915A under the constraint of a total inductance of 0.15 H, achieving an overall energy storage of 20 MJ. Multi-dimensional parameter analysis showed that with array gaps *g*_1_ = 2.44 m, *g*_2_ = 4.77 m, the critical current degradation caused by mutual inductance was effectively suppressed.

This study delivers a feasible engineering solution for high-capacity magnet design, while establishing a valuable framework and practical case study for academic research and educational applications in superconducting power technology.

## Figures and Tables

**Table 1:** Basic structural parameters of the single solenoid coil.

Parameter	Value
Superconducting core width *W_hts_*	4 mm
Encapsulated tape width *W_tape_*	5 mm
Encapsulated tape thickness *T_hts_*	0.3 mm
Tape insulation thickness *T_ins_*	0.05 mm
Total tape length *S*	5000 m
Anisotropy parameter *α*	0.88
Anisotropy parameter *B*_1_	1.59
Anisotropy parameter *γ*	3.38
Initial critical current *I_c_*_0_	1736 A
Number of *I_c_* segments per turn *n*	40
Maximum number of pancakes	100
Minimum number of pancakes	20
Maximum inner radius	500 mm
Minimum inner radius	300 mm
Outer radius limit	1000 mm
Pancake gap	1 mm

**Table 2:** Critical current, inductance, and energy storage capacity for movable pancakes per side ranging from 9 to 17.

Movable Pancakes per Side (m)	Critical Current (A)	Inductance (H)	Energy Storage (MJ)
9	866	2.77	1.04
10	880	2.75	1.07
11	886	2.74	1.07
12	899	2.71	1.09
13	900	2.71	1.10
14	915	2.67	1.12
15	917	2.64	1.11
16	915	2.62	1.10
17	923	2.57	1.09

## Data Availability

The original contributions presented in this study are included in the article. Further inquiries can be directed to the corresponding authors.

## Appendix A. Full Expressions for Core Functions $\psi_{1}$ and $\psi_{2}$

The complete definitions of the core functions $\psi_{1\;}$ and $\psi_{2}$ used in the 2D axisymmetric magnetic field model (Section 2.1) are provided here.

The function $\psi_{1\;}$ is given by:

(A1) $$\begin{array}{c} \psi_{1}\left(R_{i},R_{o},r,a\right)\\ =\frac{\mu_{0}J}{2}\left(R_{o}-R_{i}\right)-\frac{\mu_{0}Ja}{2}ln\left(\frac{R_{o}+r+\sqrt{{\left(R_{o}+r\right)}^{2}}+a^{2}}{R_{i}+r+\sqrt{{\left(R_{i}+r\right)}^{2}\;}+a^{2}}\right)\\ +\frac{\mu_{0}Jar}{2\pi }\int_{0}^{\pi }\frac{\theta sin\theta }{R_{o}-rcos\theta +X\left(R_{o},r,a,\theta \right)}\left(1+\frac{R_{o}}{X\left(R_{o},r,a,\theta \right)}\right)d\theta \\ -\frac{\mu_{0}Jar}{2\pi }\int_{0}^{\pi }\frac{\theta sin\theta }{R_{i}-rcos\theta +X\left(R_{i},r,a,\theta \right)}\left(1+\frac{R_{i}}{X\left(R_{i},r,a,\theta \right)}\right)d\theta \\ -\frac{\mu_{0}Jr}{2\pi }\int_{0}^{\pi }arctan\left(\frac{rsin\theta }{a}\right)\times \left(\frac{R_{o}}{X\left(R_{o},r,a,\theta \right)}-\frac{R_{i}}{X\left(R_{i},r,a,\theta \right)}\right)sin\theta d\theta , \end{array}$$

The function $\psi_{2\;}$ is given by:

(A2) $$\begin{array}{c} \psi_{2}\left(R_{i},R_{o},r,a\right)\\ =\frac{\mu_{0}Jr}{4}ln\left(\frac{R_{o}+r+\sqrt{{\left(R_{o}+r\right)}^{2}+a^{2}}}{R_{i}+r+\sqrt{{\left(R_{i}+r\right)}^{2}+a^{2}}}\right)+\\ \frac{\mu_{0}J}{2\pi }\int_{0}^{\pi }cos\theta (X\left(R_{o},r,a,\theta \right)-X\left(R_{i},r,a,\theta \right))d\theta \\ -\frac{\mu_{0}Jr^{2}}{4\pi }\int_{0}^{\pi }\frac{sin\theta \left(\theta +sin\theta cos\theta \right)}{R_{o}-rcos\theta +X\left(R_{o},r,a,\theta \right)}\left(1+\frac{R_{o}}{X\left(R_{o},r,a,\theta \right)}\right)d\theta \\ +\frac{\mu_{0}Jr^{2}}{4\pi }\int_{0}^{\pi }\frac{\left(\theta +sin\theta cos\theta \right)sin\theta }{R_{i}-rcos\theta +X\left(R_{i},r,a,\theta \right)}\left(1+\frac{R_{i}}{X\left(R_{i},r,a,\theta \right)}\right)d\theta \end{array}$$
